# Gene-based molecular analysis of *COX1* in *Echinococcus granulosus* cysts isolated from naturally infected livestock in Riyadh, Saudi Arabia

**DOI:** 10.1371/journal.pone.0195016

**Published:** 2018-04-18

**Authors:** Dina M. Metwally, Latifa E. Qassim, Isra M. Al-Turaiki, Rafa S. Almeer, Manal F. El-Khadragy

**Affiliations:** 1 Zoology Department, Faculty of Science, King Saud University, Riyadh, Kingdom of Saudi Arabia; 2 Parasitology Department, Faculty of Veterinary Medicine, Zagazig University, Zagazig, Egypt; 3 Information Technology Department, College of Computer and Information Science, King Saud University, Riyadh, Kingdom of Saudi Arabia; 4 Department of Zoology and Entomology, Faculty of Science, Helwan University, Cairo, Egypt; University of Minnesota, UNITED STATES

## Abstract

The diversity and importance of *Echinococcus* species in domesticated animals in Saudi Arabia are poorly understood. In this study, 108 singular (hydatid) cysts were collected from goats (n = 25), sheep (n = 56) and camels (n = 27). DNA was extracted from the protoscoleces of individual fertile cysts and used for polymerase chain reaction (PCR) amplification of mitochondrial subunit 1 of the *cytochrome c oxidase 1* (*cox1*) gene. Amplicon sequencing results revealed the presence of *Echinococcus granulosus* sensustricto (s.s.) (genotypes G1–G3) in 16 of the17 sheep cysts and 2 of the 27 camel cysts.of these samples, 18 (2 camel and 16 sheep) were divided into genotypes G1, G2, and G3.

## Introduction

Cystic echinococcosis (CE) is a prevalent zoonotic disease caused by parasitic infection [1].CE is most widespread in rural areas with few to no hygiene facilities and poor living conditions where humans, dogs, and animals reside in close proximity [2]. The parasitic lifecycle includes eggs that are passed by definitive hosts (canids) harboring adult worms.The eggs subsequently develop to reach the cystic stage after ingestion by an intermediate host [3].CE affects millions of people worldwide and is highly endemic in Mediterranean coastal regions, South America, Eastern Europe, the Middle East, East Africa, China, Central Asia, and Russia [4]. The disease is also estimated to cause yearly financial losses of several billion dollars in domesticated animals.This is partly the result of low eradication rates, mortality in infected animals, and the need to discard the contaminated organs of slaughtered animals [5].Genetic examinations of hydatid cysts innumerous geographical areas have led to the discovery of ten genotypes: G1–G10 [6–9]. *Echinococcus granulosus* sensulato was recently categorized into four distinct species: *E*. *granulosus* sensu stricto (s.s.) (G1–G3), *Echinococcus ortleppi* (G5), *Echinococcus equinus* (G4) and *Echinococcus canadensis* (G6–G10) [10, 11]. Although the status of genotype G9 remains uncertain [12], it may be similar to the genotype found in pigs (G7) [13]. The *E*. *granulosus* s.s. G1 genotype (found in sheep) has been implicated innumerous human CE cases. In addition, human infections have been described for every genotype except G4 [13,14]. A phylogenetic examination of the *cytochrome c oxidase 1*(*cox1*) gene indicated that the primary strains observed in sheep (G1) and buffalo (G3) cycle among domesticated animals and have adapted to goats, camels and cattle. Human infections have been linked to the G1 basic sheep genotype of *E*. *granulosus*, suggesting that these strains are highly capable of engaging in zoonotic exchange [15]. Better characterization of *Echinococcus* species may improve the development and advancement of control measures, indicative tests, and treatment options [11,16].Studies performed in Saudi Arabia have investigated the general prevalence of *Echinococcus* [17–20],although little information is available regarding the zoonotic potential of this parasite. In the present study, we analyzed hydatid cysts using a polymerase chain reaction (PCR) sequencing strategy to evaluate the mitochondrial *cox1* gene in domesticated animals (sheep and camels) in Saudi Arabia with the aim of expanding what is known about *E*. *granulosus*.

## Materials and methods

### Ethics statement

The Institutional Committee of Post-graduate Studies and Research at King Saud University (Saudi Arabia) approved this study. Hydatid cysts were collected by veterinarians during post-mortem inspections of slaughtered animals performed at the Al-Sada Abattoir in Riyadh, Saudi Arabia, in April and October 2016. Official approval of the use of hydatid cysts (for research purposes only) was obtained from the university as well as the abattoir veterinarians.

### Sample collection

A total of 108 hydatid cysts were collected from sheep (n = 56), goats (n = 25) and camels (n = 27).Each cyst was considered an isolate, and all cysts were isolated from the liver. To determine whether the cysts were fertile, the contents of each cyst were aseptically aspirated and dispensed into sterile Petri dishes, and the presence of protoscoleces (fertile cysts) was visually determined. Protoscoleces were specifically collected from single fertile cysts under nuanced aseptic conditions and subsequently washed as many as three times using a sterile saline solution before being fixed in 95% ethanol.

### DNA extraction and PCR amplification

Protoscoleces obtained from cysts were washed with distilled water and ethanol before they were centrifuged. Genomic DNA (gDNA) was then extracted using a High Pure PCR Template Preparation Kit (Qiagen GmbH, Hilden, Germany, Cat. No.51304).The mitochondrial *cox1* gene was amplified with the reverse primer 5’-TAAAGAAAGAACATAATGAAAATG-3’[6] and the forward primer 5’- TTTTTTGGGCATCCTGAGGTTTAT-3’in a 40-μl reaction mixture containing 8 μl of master mix, 25.6 μl of deoxynucleotides (dNTPs), 2.4 μl of primers and 4 μl of DNA template. The PCR program consisted of an initial denaturation step at 94°C for 5 minutes, followed by 40 cycles of denaturation at 94°C for 45 seconds, annealing at 50°C for 45 seconds, and extension at 72°C for 10 minutes, and a final extension step at 72°C for 10 minutes. The PCR products were analyzed by 1% agarose gel electrophoresis.

### DNA sequencing and phylogenetic analysis

The sequences of the forward strands were aligned using ClustalW [11] implemented in Geneious software version 10.0.7 [21]. Multiple sequence alignment was performed once for each group of samples (camel, sheep, and a combination of all samples). Multiple sequence alignment included the genotypes G1, G2, and G3, and G1 was set as the reference sequence. Low-quality sequence ends were trimmed to obtain better results. A phylogenetic tree was generated from the trimmed sequences obtained in this study in addition to standard sequences for *E*. *granulosus* genotypes (G1–G10) and other *Echinococcus species*. *Taenia saginata* was used as the out-group (Table 1). The neighbor-joining method [22] was used with the *Tamura Nei* model to generate the phylogenetic tree. The bootstrap method was used for resampling with the number of replicates set to 1000.

**Table 1:** *E*. *granulosus* haplotypes and reference sequences utilized for phylogenetic analysis of partial *cox1* sequences.

Haplotype, genotype or species	Host	Accession number (*cox1*)	Reference
Echs1	Sheep	---------	The present study
Echs2	Sheep	---------	The present study
Echs3	Sheep	---------	The present study
Echs4	Sheep	---------	The present study
Echs5	Sheep	---------	The present study
Echs6	Sheep	---------	The present study
Echs7	Sheep	---------	The present study
Echs8	Sheep	---------	The present study
Echs9	Sheep	---------	The present study
Echs10	Sheep	---------	The present study
Echs11	Sheep	---------	The present study
Echs12	Sheep	---------	The present study
Echs13	Sheep	---------	The present study
Echs14	Sheep	---------	The present study
Echs15	Sheep	---------	The present study
Echs16	Sheep	---------	The present study
Echs17	Sheep	---------	The present study
Echs18	Sheep	---------	The present study
Echc1	Camel	---------	The present study
Echc2	Camel	---------	The present study
G1	Sheep	U50464	[23]
G2	Sheep	M84662	[6]
G3	Buffalo	M84663	
G4	Horse	M84664	
G5	Cattle	M84665	
G6	Camel	M84666	
G7	Pig	M84667	
G8	Moose	AB235848	[10]
G10	Reindeer	AF525457	[9]
*E*. *multilocularis*	Human	M84668	[6]
*E*. *multilocularis*	Rodent	M84669	[6]
*E*. *vogeli*	Rodent	AB208064	[10]
*E*. *shiquicus*	Pika	M84670	[6]
*E*. *oligarthrus*	Rodent	M84671	[6]
*E*. *felidis*	Lion	EF558356	[24]
Outgroup: *Taeniasaginata*	Cattle	AB465239	[25]

## Results

### Hydatid cyst collection

After the collected cysts were assessed, 17 of the 56 cysts extracted from sheep and 2 of the 27 cysts collected from camels were found to be fertile. All 25 cysts derived from goats were infertile. PCR amplification and DNA sequencing were performed for all fertile cysts. Unfertile cysts were not processed for molecular analysis.

### Amplification of the mitochondrial *cox1* gene

**Fig 1:**
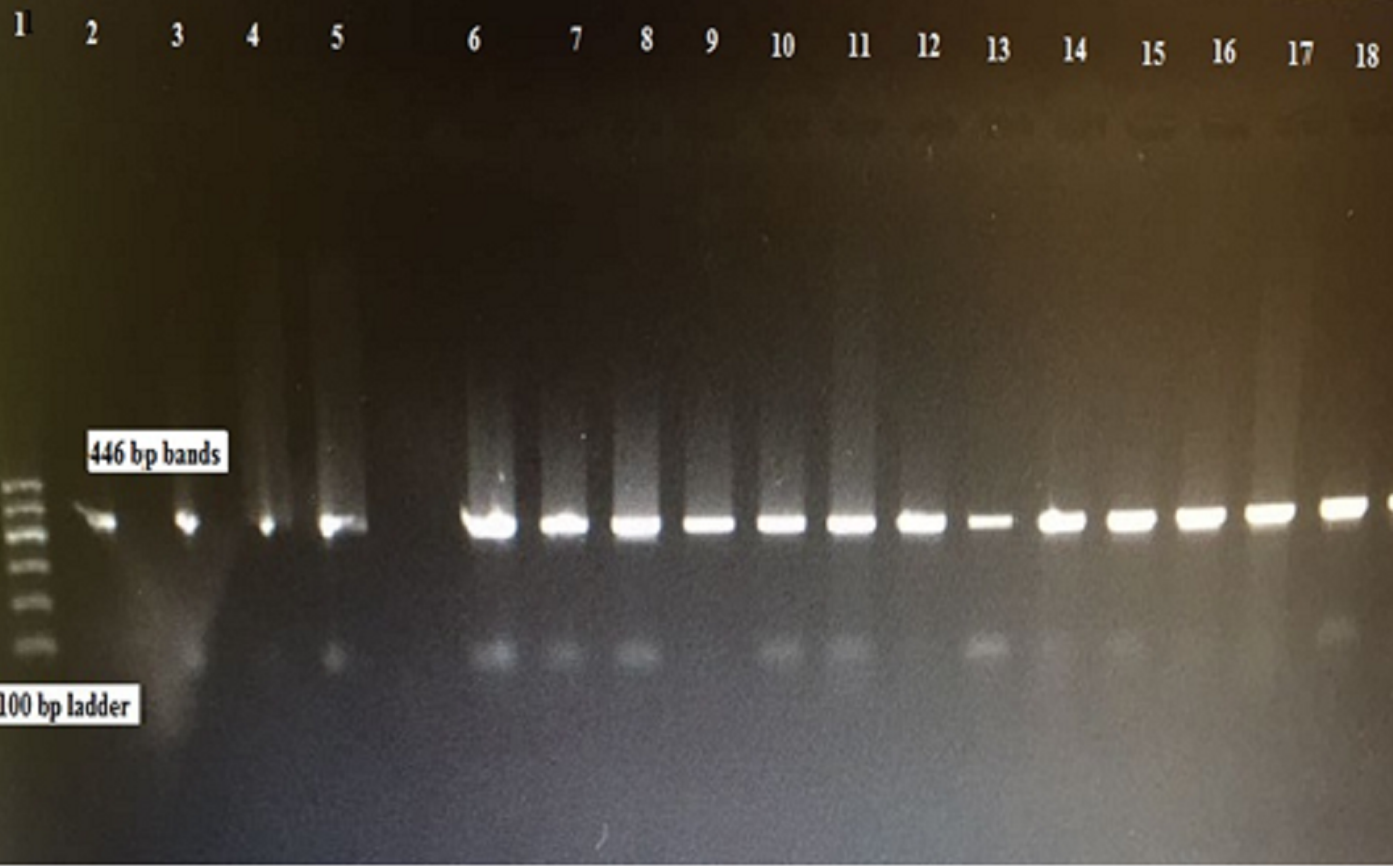
Agarose gel (1.0%) electrophoretogram with 100-bp DNA ladder. PCR analysis of the *cox1* gene revealed a 446-bp band derived from different sheep hydatid cyst isolates (2–18).

**Fig 2:**
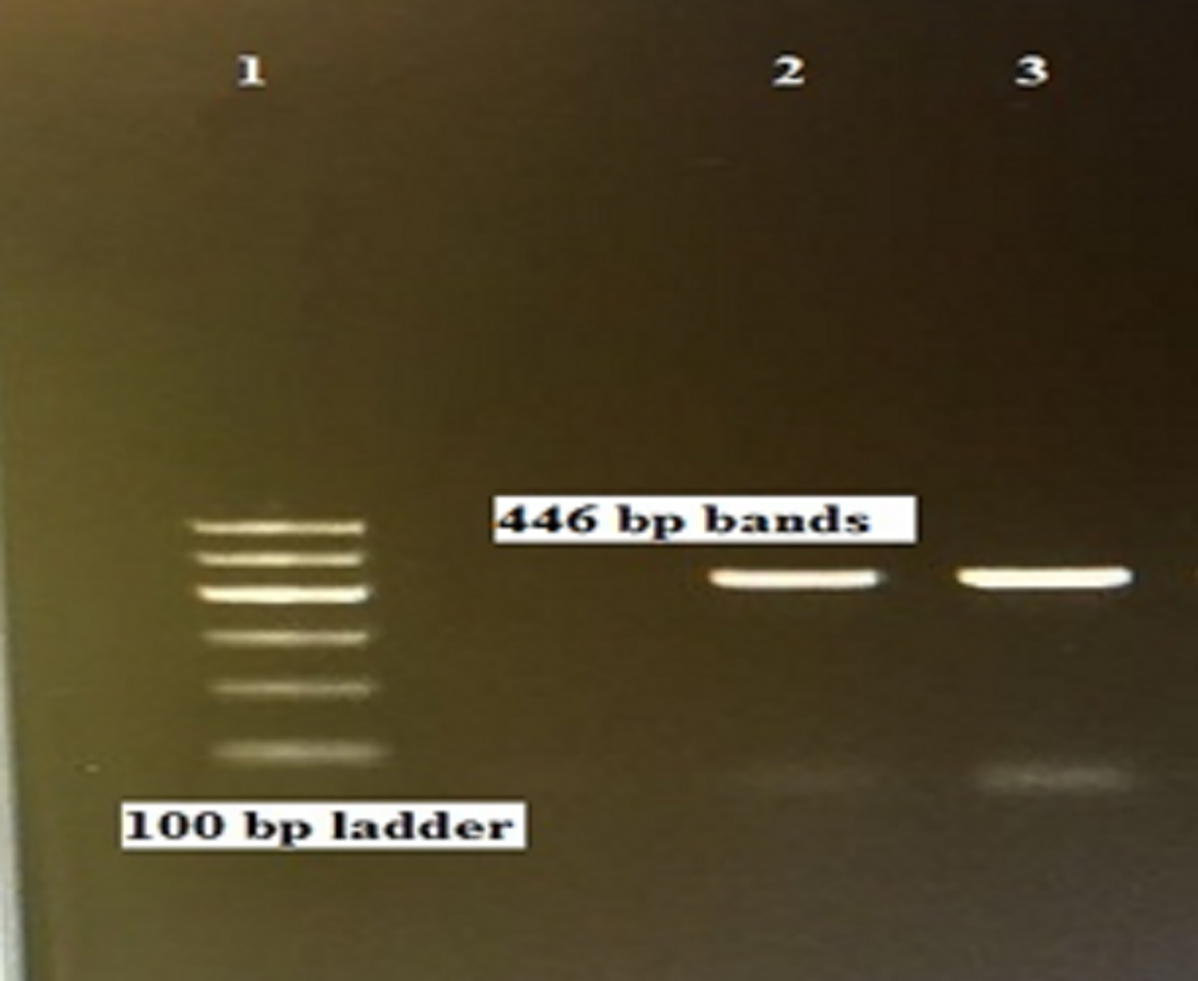
Agarose gel (1.0%) electrophoretogram with 100-bp DNA ladder. PCR analysis of the *cox1* gene revealed a 446-bp band derived from different camel hydatid cyst isolates (2, 3).

A 446-bp fragment of the *cox1* gene was PCR amplified from gDNA extracted from each of the fertile hydatid cysts (Figs 1 and 2).

#### Camel samples

**Fig 3:**
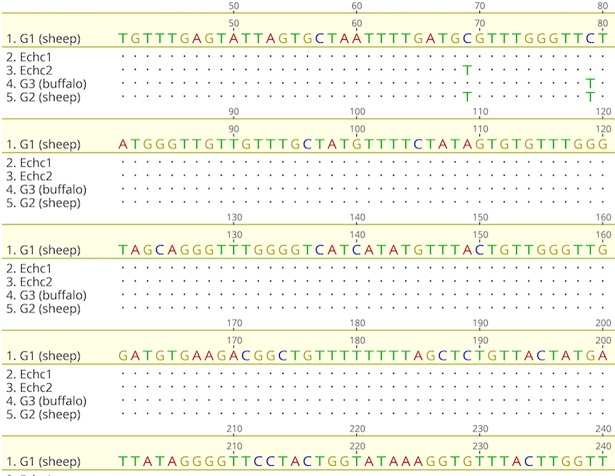
Multiple sequence alignment of two camel samples and genotypes G1, G2, and G3.

**Fig 4:**
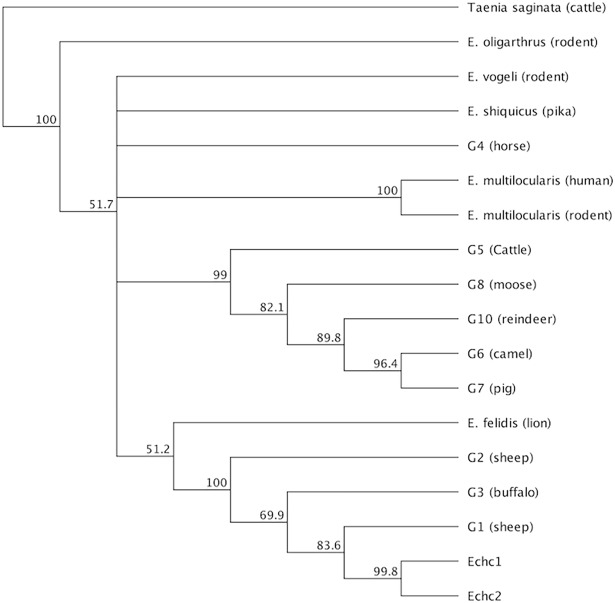
Phylogenetic tree of the sequences of camel samples from Saudi Arabia and reference sequences obtained from previous studies.

A portion of the multiple sequence alignment of camel samples *Echc1* and *Echc2* with genotypes G1, G2, and G3 is shown in Fig 3. We found a one-nucleotide substitution (C to T) in one sample at position 69 of the reference sequence. Fig 4 shows the phylogenetic tree of the camel samples and reference sequences. The results indicate that G1 was the only genotype found in the camel samples. Table 2 shows the genetic distance matrix.

**Table 2:** Genetic distance matrix based on the Tamura Nei model for the camel samples and genotypes G1, G2, and G3.

	Echc1	Echc2	G1 (sheep)	G2 (sheep)	G3 (buffalo)
**Echc1**		0.01	0.019	0.017	0.014
**Echc2**	0.01		0.016	0.014	0.017
**G1 (sheep)**	0.019	0.016		0.008	0.006
**G2 (sheep)**	0.017	0.014	0.008		0.003
**G3 (buffalo)**	0.014	0.017	0.006	0.003	

#### Sheep samples

**Fig 5:**
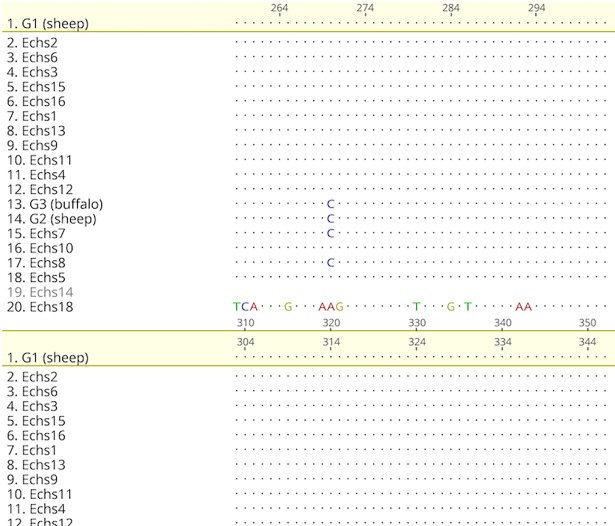
Multiple sequence alignment of sheep samples and genotypes G1, G2, and G3.

**Fig 6:**
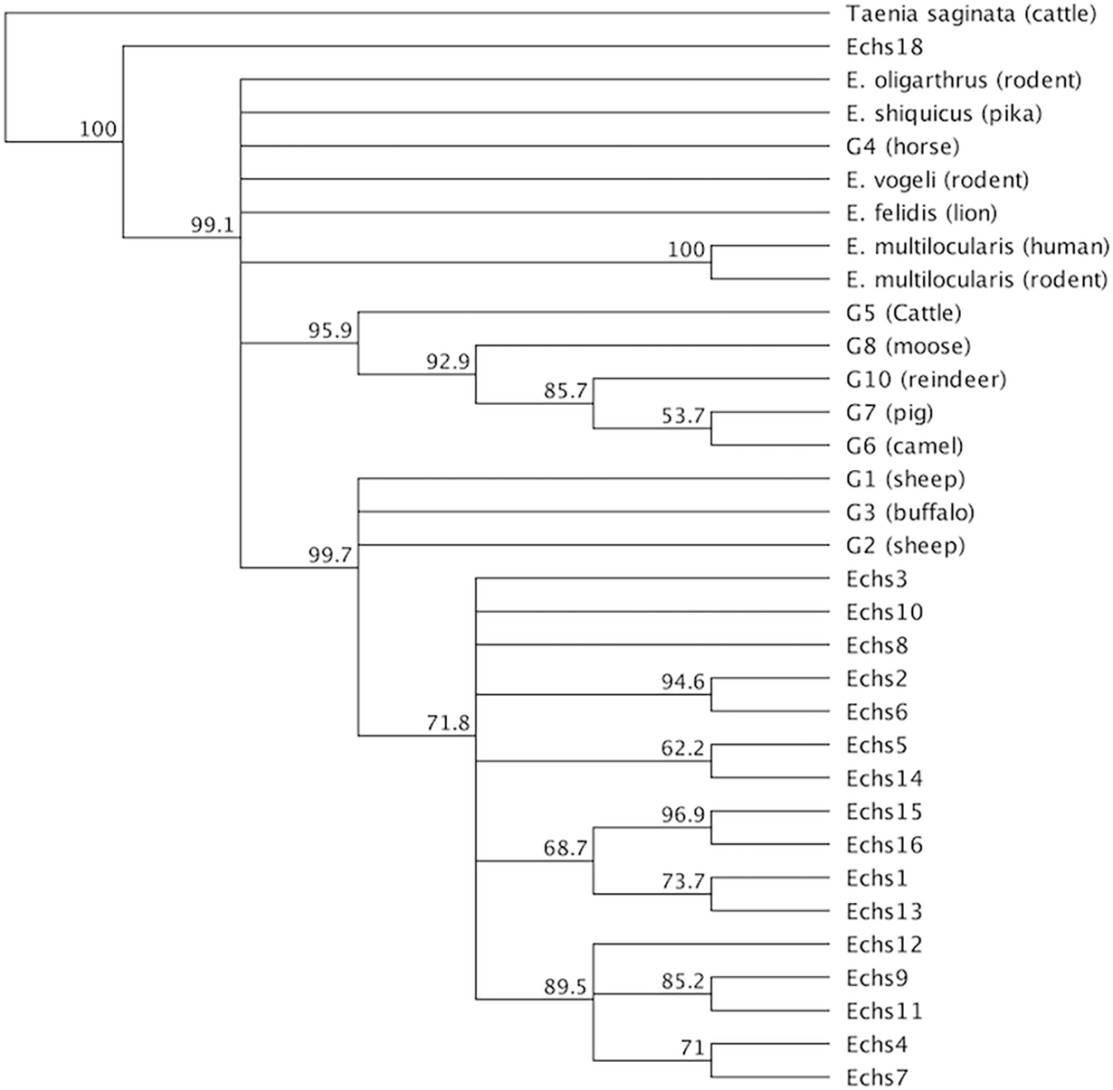
Phylogenetic tree of the sequences of 17 sheep samples and reference sequence obtained from previous studies.

A portion of the multiple sequence alignment of the 17 sheep samples with genotypes G1, G2, and G3 is shown in Fig 5. We found the following one-nucleotide substitutions: in 7 samples, C to T at position 69 of the reference sequence (SNP analysis indicates that this change is a transition SNP);in 1 sample, C to T at position 79;in 1 sample, A to T at position 98; in 1 sample, G to T at position 100;in 1 sample, T to G at position 101;in 1 sample, C to T at position 105;in 1 sample, C to T and C to A at position 124; and in 2 samples, T to C at position 270. Fig 6 shows the phylogenetic tree of the sheep samples and reference sequences. The two samples, *Echs15* and *Echs16*,formed a clade with bootstrap support of 96.9%. The pair *Echs2* and *Echs6* also formed a clade with bootstrap support of 94.6%.Similarly, *Echs9* and *Echs11* formed a clade with bootstrap support of 85.2%. *Echs1* and *Echs13* formed a clade with bootstrap support of 73.7%. *Echs4* and *Echs7* formed a clade with bootstrap support of 71%.

Overall, the results grouped 16 out of the 17 samples in a clade with genotypes G1, G2, and G3, with bootstrap support of 99.7%. Sample *Echs18*seemed less related to the other sheep sequences and more related to the outgroup.

#### Sheep and camel samples

**Fig 7:**
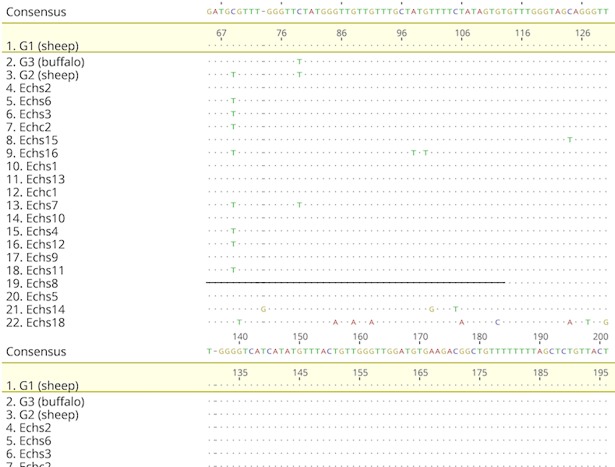
Multiple sequence alignment of sheep and camel samples and reference sequences obtained from previous studies.

**Fig 8:**
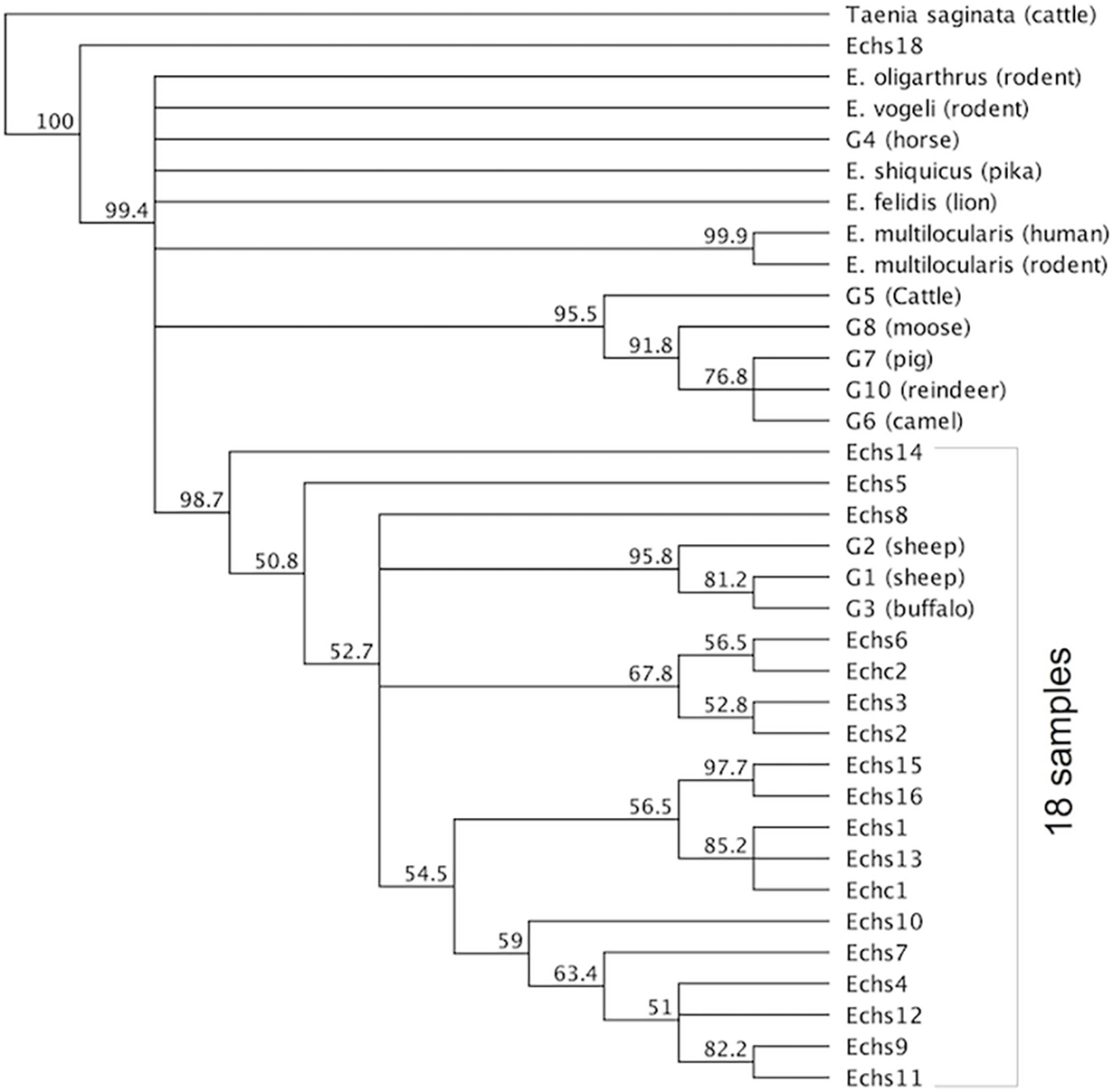
Phylogenetic tree of all samples and reference sequences obtained from GenBank.

The multiple sequence alignment of the sheep and camel samples with genotypes G1, G2, and G3 is shown in Fig 7. We found the following one-nucleotide substitutions: C to T at position 69of the reference sequence (SNP analysis indicates that this change is a transition SNP appearing in 8 of the samples, including one camel sample); C to T at position 79; A to T at position 98; G to T at position 100; T to G at position 101; C to T at position 105; and T to C at position 270. Fig 8 shows the phylogenetic tree of the sheep samples and reference sequences.

Two sheep samples, *Echs1* and *Echs13*, formed a clade with camel sample *Echc1*,with bootstrap support of 85.2%.Overall, the results grouped18of the samples (2 camel and 16 sheep) in a clade with G1, G2, and G3, with bootstrap support of 98.7%.

## Discussion

The results of this study show that as many as three separate genotypes of *E*. *granulosus*, including G1, G2, and G3, are present in Saudi Arabia and that they are frequently found in sheep, cattle, and buffalo, respectively. These findings are similar to those of studies performed in Iran suggesting that G1 remains the most prevalent *E*. *granulosus* genotype in livestock [26] and that G2 is the second most common genotype in both cattle and sheep [27,28]. A study performed in Turkey assessed nearly 208 isolates (19 cattle, 179 sheep, 7 goats, 1dog and 1 camel) and detected only the G1 genotype. However, that study mainly utilized PCR-based restriction fragment length polymorphism (PCR-RFLP) targeted to the internal transcribed spacer (ITS)-1 region of ribosomal DNA and performed a *cox1* sequence analysis for only a handful of isolates [29]. Genotype G3 of *E*. *granulosus* has been isolated from cattle, buffalo and sheep in Turkey, Italy, India and Chile [30–34], and studies worldwide have demonstrated many similarities between the distributions of genotypesG1 and G3. For example, in southern Italy, an investigation of 48 wild water oxen detected G3 in 31.25% and G1 in 68.75% of CE cases [35]. Other studies have reported comparable patterns with varying proportions of G1 and G3. For example, in a study of 112 cattle and sheep performed in Turkey, 95.5% of CE cases were G1,while 4.5% were G3 [36]; in a study of 30 cows, sheep and humans performed in Tunisia, 93.3% of CE cases were G1,and 6.7% were G3 [37]; in a study of 80 cattle and water buffaloes performed in Italy, 78.75% of CE cases were G1,and 12.5% were G3 [30]; in a study of 38 animals performed in southeastern Iran, 73.7% of CE cases were G1,and13.2% were G3 [38]; and in a study of 18 humans and dogs performed in southern Brazil, 77.8% of CE cases were G1,and 11.1% were G3 [39].Another study identified four different *E*. *granulosus* genotypes, G1, G2, G3, and G5, in 46 household animals in India [40]. Of these genotypes, G3 was the most prevalent, accounting for 63% of the CE cases, whereas G1 was observed in only six (13%) cases. Furthermore, a unique situation was observed in a study of 19 different hydatid cyst samples extracted from camels in central Iran. In that study, the *nad1* and *pcox1*genes were sequenced, and the G3 genotype was identified in 42.1%, the G6 genotype in 31.6% and the G1 genotype in 26.3% of the cases [41]. The camel genotype clearly differs from the genotypes found in other domestic animals throughout numerous regions in Iran. While only the G6 genotype has been found in some African countries [42–44], no G6 isolates were identified in the current study. Several studies based on *cox1* analysis have also demonstrated different haplotypes within genotypes G3and G1 in various hosts [34, 45–47]. For example, one study of 112 cattle and sheep hydatid cyst isolates (in Turkey) identified 5 G3 isolates, 107 G1 isolates, and 5 unique haplotypes [37]. In the present study, 18 samples (2 camel and 16out of 17 sheep) were grouped with genotypesG1, G2, and G3, and these isolates were compared with other genotypes. The horizontal branches in a phylogenetic tree indicate genetic distances (i.e., the amount of genetic change), while longer horizontal branches are associated with greater divergence. Our analysis of the combined sheep and camel samples revealed the following one-nucleotide substitutions: C to T at position 69, C to T at position79, A to T at position 98, G to T at position 100, T to G at position 101, C to T at position 105, and T to C at position 270 of the reference sequence. The substitution at position 69 of the reference sequence was identified in 8 of the samples, including one camel sample. A phylogenetic tree constructed using all the isolates (Fig 8) showed that the G1, G2 and G3 genotypes comprise a deeply related complex that is distinct from other genotypes (G4 to G10),as previously described by other investigators [35,47,48]. Because most of the sheep used by humans in Saudi Arabia are imported from Sudan, these animals are the likely source of the *E*.*granulosus* s.s. observed in Saudi Arabia. These harmful cysts are economically important because of their impact on animal health, and the findings of the present work are therefore valuable because they establish the exact genotypes present in each species; this will enable appropriate preventative measures and therapeutic strategies to be implemented in the various animal populations affected by CE. Obtaining additional isolates from other hosts, such as humans and stray canines, and from other geographic areas may be necessary to increase our understanding of the distribution of CE in Saudi Arabia.

## Supporting information

S1 Dataset All samples in the present study provided in two formats: FASTA and chromatogram. (ZIP)
